# Rivers and roads, silence and songs: female crickets respond similarly to conspecific male song in natural and anthropogenic soundscapes

**DOI:** 10.1242/jeb.250817

**Published:** 2026-01-26

**Authors:** Erik A. Etzler, Hannah M. ter Hofstede, John M. Ratcliffe

**Affiliations:** ^1^ Department of Ecology and Evolutionary Biology, University of Toronto, Toronto, ON, Canada M5S 3B2; ^2^ Department of Biology, University of Toronto Mississauga, Mississauga, ON, Canada L5L 1C6; ^3^ Department of Integrative Biology, University of Windsor, Windsor, ON, Canada N9B 3P4

**Keywords:** Anthropocene, Orthoptera, Road noise, Hearing, Neuroethology, Mate finding

## Abstract

Many studies have demonstrated that anthropogenic noise affects animals' auditory perception of salient stimuli. Few have tested whether these effects are different from those experienced in nature. We tested the ability of female field crickets, *Teleogryllus oceanicus*, to phonotactically locate a speaker playing conspecific male song in four acoustic backgrounds: silence, road noise, river noise and heterospecific song. Crickets approaching conspecific song paused more frequently in river noise and heterospecific song treatments compared with silence or road noise. We also recorded auditory interneuron (AN1 and AN2) activity under the first three acoustic background treatments to construct and compare treatment-specific audiograms and interneuron responses to conspecific song. We found little difference in activity, other than that AN2 thresholds for 6 kHz sounds (the tested frequency closest to male song) were highest in river noise, while heterospecific song increased baseline AN2 activity and reduced AN2 activity to conspecific song onset. Our results suggest road noise is not necessarily a greater disturbance than river noise.

## Teaser

**Summary:** Crickets perceive and react to traffic noise in the same way as they do to natural sources of noise in the environment.

## INTRODUCTION

We are increasingly aware that anthropogenic noise can harm other animals. Its potential to interfere with communication, foraging and vigilance has been demonstrated in birds (Allen et al., 2021; Habib et al., 2007; Mason et al., 2016), mammals (Bunkley and Barber, 2015; Siemers and Schaub, 2011), anurans (Roca et al., 2016) and arthropods (Gordon and Uetz, 2012; Schmidt et al., 2014; Wale et al., 2013). Insects are important indicators of ecosystem health (Longcore, 2003; Sánchez-Bayo and Wyckhuys, 2019) and are increasingly used to study the effects of anthropogenic noise on wildlife. They are well suited to studying the effects of noise pollution because many use sound for communication and can be raised in large numbers in the laboratory (Morley et al., 2013). Acoustic communication is especially widespread in orthopterans (i.e. crickets, katydids and grasshoppers; Song et al., 2015), rendering them vulnerable to acoustic interference and thus an ideal system to examine the effects of anthropogenic noise (Morley et al., 2013). To date, the effect of anthropogenic noise on orthopterans remains unclear because reported results are often conflicting. That is, across species, real-time exposure to traffic noise during phonotaxis has been found to increase the time needed to locate mates (Bent et al., 2021; Schmidt et al., 2014) or have no effect at all (Costello and Symes, 2014; Gurule-Small and Tinghitella, 2018). In field crickets (Gryllinae, Gryllidae) specifically, long-term exposure to traffic noise has been shown to increase (Etzler et al., 2025a; Gurule-Small and Tinghitella, 2018) or decrease (Rebar et al., 2022) time to locate mates. There are also indications of behavioural compensation in the timing or spectra in the songs of orthopteran populations that live near to roads (Costello and Symes, 2014; Gallego-Abenza et al., 2020; Lampe et al., 2014; Orci et al., 2016).

However, a world without humans would still be a noisy one. Natural environmental noise has long interfered with acoustic communication by decreasing signal-to-noise ratio, limiting the use of certain frequencies, or spatiotemporally constraining communication (Brumm and Slabbekoorn, 2005). Whether or how anthropogenic noise differs in its effects from natural sounds such as rivers, wind and acoustic signals of sympatric animal species has not been extensively studied. Further, orthopterans have evolved mechanisms to deal with many natural interference noises, including other orthopteran species (Gomes et al., 2021; Schmidt and Balakrishnan, 2015). That said, orthopterans often sing during quieter periods, apparently to avoid noise (Greenfield, 1988, 1993), and some physically change ear opening shape to limit interference (Römer et al., 1989; Römer and Bailey, 1998). White noise does not reduce the accuracy of song localization in grasshoppers (Reichert, 2015) and may even make attractive males more attractive still (Einhäupl et al., 2011). To the best of our knowledge, no study has yet compared the effects of traffic noise with those of naturally occurring sounds on hearing and phonotactic mate searching behaviour of female orthopterans.

In the study we report here, we sought to determine whether detection and localization of male conspecific song by adult female crickets differs in traffic noise compared with natural loud, persistent sounds in the environment by comparing phonotaxis with conspecific male song and the activity of two auditory interneurons (AN1 and AN2) under different background acoustic treatments. By using both behavioural and neural metrics, as well as directly comparing traffic noise with non-human noises, we hope to better understand why previous findings have so often been contradictory. Detection (being aware of the presence of a stimulus) and localization (being able to locate and reach the stimulus) are both necessary components of mating in our study system. By examining how quickly female crickets can reach a simulated male and the relative activity of auditory neurons, we can begin to separate the two effects. We used the field cricket *Teleogryllus oceanicus* as our study subject specifically because this species' acoustically evoked behaviour and auditory neural activity have been extensively studied. The auditory interneurons, AN1 and AN2, are involved in detecting conspecific calling song and predator sounds (Fullard et al., 2010; Nolen and Hoy, 1984; Schöneich, 2020; ter Hofstede et al., 2009).

The AN1 interneuron is involved in detection and encoding of conspecific song. However, the signal to noise ratio in our recordings was not sufficient to reliably identify AN1 spikes. The more easily recorded AN2 is broadly tuned and responds to high-frequency predator cues (e.g. echolocating bats) and male song in a way that also positively correlates with phonotaxis (Jeffery et al., 2005; Samuel et al., 2013; Schildberger and Hörner, 1988; Schöneich, 2020; Stout et al., 2011). In addition to its main function in negative phonotaxis for predator avoidance (Pollack, 2015), AN2 had also be speculated to potentially influence phonotaxis towards calling song at high amplitudes (e.g. Schildberger and Hörner, 1988). We measured both interneurons in a set up that mimicked close-range detection and localization behaviours in females searching for males. Specifically, we compared behaviours related to phonotaxis in four background treatments: traffic noise, silence, river noise and heterospecific song (of *Teleogryllus commodus*). These are acoustic conditions that *T. oceanicus* would encounter across their geographic range. We predicted that river noise and traffic noise would have a similar frequency content and thus that we would observe a similar decline in song localization performance under these acoustic conditions, as previously observed for road noise by Gurule-Small and Tinghitella (2018), compared with silence or *T. commodus* song. We predicted no significant difference in performance between silence and *T. commodus* song because *T. oceanicus* possesses spectral and temporal filters to discriminate between its own and sympatric *T. commodus* song (Bailey et al., 2017; Hoy et al., 1982).

## MATERIALS AND METHODS

### Sound recordings

**Fig. 1.**
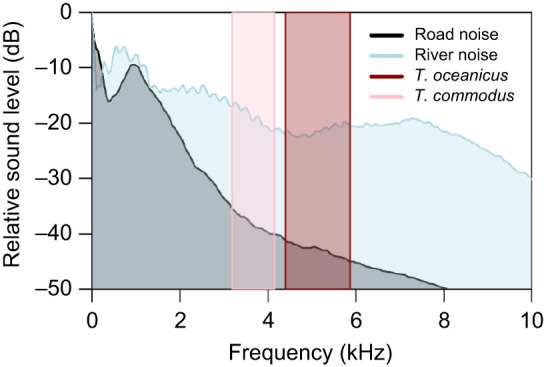
**Relative energy across frequencies for noise and signals used in this study.** Power spectra are shown for 3 min of traffic noise recorded at a highway intersection in Mississauga, ON, Canada (black), and 3 min of river noise recorded at the Credit River in Erindale Park in Mississauga, ON, Canada (blue). Vertical shaded bars represent the range of frequencies in the calling songs of *Teleogryllus oceanicus* (red) and *Teleogryllus commodus* (pink) (lower and upper limits measured at −35 dB below the frequency with most energy).

**Fig. 2.**
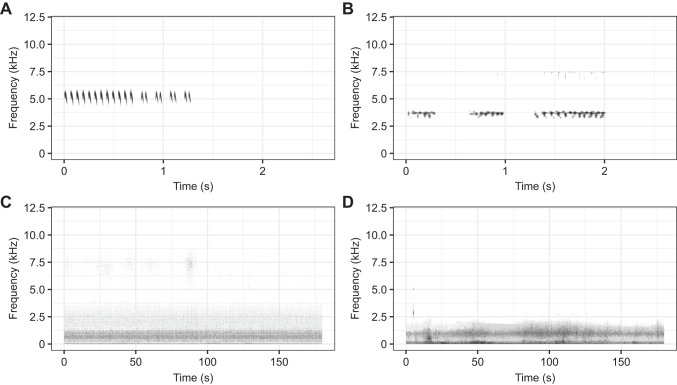
**Spectrograms of the playbacks used in this study.** Spectrograms are shown for one full repeat of the songs of (A) *T. oceanicus* and (B) *T. commodus* as well as one full minute of the playbacks of (C) river noise recorded at the Credit River in Erindale Park in Mississauga, ON, Canada, and (D) traffic noise recorded at a highway intersection in Mississauga, ON, Canada.

As reported in a related study (Etzler et al., 2025a), we recorded traffic noise at the Hurontario and Queen Elizabeth Way on-ramp and river noise at the Credit River in Erindale Park (both locations in Mississauga, ON, Canada) using a Song Meter Mini Bat (Wildlife Acoustics, Maynard, MA, USA) sampled at 24 kHz (Figs 1 and 2). We used a downloaded (Orthoptera Species File Website; Cigliano et al., 2024) and modified (Gurule-Small and Tinghitella, 2018; Bailey, 2008) recording of one full repeat of a calling song of *T. oceanicus* (sampled at 96 kHz) and one full repeat of a downloaded calling song of *T. commodus* from the Commonwealth Scientific and Industrial Research Organisation (CSIRO) website (sampled at 22 kHz) (Figs 1 and 2).

### Animals and animal rearing treatments

We used *Teleogryllus oceanicus* (Le Guillou 1841) crickets from a colony maintained at the University of Toronto Mississauga (UTM). *Teleogryllus oceanicus* can be found across Oceania, in coastal Australia and other coastal islands (Zuk et al., 2001). Our *T. oceanicus* colony was established in 2005 using eggs collected from wild-caught females from Mo'orea, French Polynesia, and from Darwin, NT, Australia. At both of these locations, the insects were caught on maintained grass fields near roads (e.g. park lawns). Laboratory populations derived from these two locations were combined in 2011. Crickets were hatched and reared in plastic boxes (width: 35 cm, length: 5 cm, height: 40 cm), partially filled with cardboard egg cartons and provided with food (1:1 ratio of cat and fish food) and water *ad libitum*. Crickets were kept at 23°C on a 14 h/10 h light/dark cycle and checked daily to identify females.

When they reached their penultimate instar, females were taken from their box and placed in individual containers. These containers consisted of a closed-top aluminium mesh cylinder (height: 10 cm, diameter: 9.5 cm) placed over an upside-down clear plastic container. Each container also contained the same food blend, water and an overturned egg carton cup as a shelter. These containers were kept in a custom-made acoustic chamber (width: 1.73 m, length: 2.03 m, height 2.03 m) lined with acoustic foam (ceiling and walls) with a floor of corkboard over carpet. The chamber contained one lamp (OttLite T81G5T-SHPR 18 W, Tampa) on a 14 h/10 h light/dark cycle and the chamber's temperature ranged from 23 to 25°C. One hundred and one female crickets were collected, of which 96 reached sexual maturity. Each cricket was tested once for this study. Thirty-nine of these crickets were also used in Etzler et al. (2025a). Crickets used in both studies were presented with the behavioural treatments in a randomized order, but each represented only a single data point in each study.

### Experimental trials

#### Behaviour

To compare the effect of the background acoustic treatments on female phonotaxis, we randomly assigned crickets at 14 days after final moult to an experimental background noise treatment of (i) traffic noise, (ii) river noise, (iii) *T. commodus* song or (iv) silence [speaker on but no sound, ambient sound level in the room was <30 dBC (1 s time weighting); Etzler et al., 2025a] during male song playback. In the centre of the experimental trial room (height: 2.3 m, width: 2.6 m, length: 4.6 m), we placed a circular arena made of cork (1.22 m diameter, 6.25 mm thick) on the floor marked with a circle of 1.2 m diameter, dividing the circle into eight segments of equal arc (45 deg). For playback of *T. oceanicus* male song, we placed a focal speaker (Vifa ultrasonic dynamic speaker, Avisoft Bioacoustics, Berlin, Germany) on the edge of the arena at the dividing line between two of the eight segments; the particular dividing line was chosen at random for each trial. The second focal speaker was placed on the direct opposite end of the arena from the first (i.e. at 180 deg from the first). Each focal speaker was flanked by two paired speakers (Companion 20 speakers, Bose, MA, USA) 25 cm to either side of it along the circumference of the arena. These flanking speakers were used to broadcast the background noise treatments. We used a Shure 57 instrument microphone (Shure, Eppingen) (flat <150 Hz to >15 kHz) to calibrate our Bose Companion 20 speakers and found them to be relatively flat (±4 dB) from 150 Hz to 15 kHz (Etzler et al., 2025a). Focal speakers exclusively played cricket song (or were silent), while flanking speakers played one of four background acoustic treatments. All speaker membranes faced the centre of the arena.

For each trial, we placed an adult female cricket in the centre of the arena under an egg carton cup and then covered the cup with a darkened plastic container to confine the cricket. The four flanking speakers played: a 3 min repeating segment of traffic noise, selected for its low variability in amplitude (∼70 dBA average, 68–78 dBA range); a 3 min repeating segment of river noise (∼70 dBA average, 69–71 dBA range); a 2.5 s repeating *T. commodus* song phrase (∼70 dBA average, 68–72 dBA range); or a silent sound file. In all background acoustic treatments, we randomly selected one of the two focal speakers to play the modified calling song of *T. oceanicus* with an amplitude of ∼70 dBA at centre of the arena. These dBA measurements were taken from a REED sound level meter (R8060, REED Instruments) at a 125 ms time weighting, which had been calibrated using a Brüel and Kjær calibrator Type 4231 calibrator (1 kHz tone, 94 dB SPL relative to 20 µPa) with its ½ inch microphone adapter in place. We determined the REED sound level meter was measuring dBA accurately at 1 kHz for 125 ms and 1 s time weightings (i.e. as 94 dB). We conducted the preceding calibration routine of experimental set-up to best match the dBA values of road noise and cricket song reported by Gurule-Small and Tinghitella (2018) in their phonotaxis experiments using *T. oceanicus* and which inspired the design our own experiments.

As an additional and more conventional check on playback sound levels, we also recorded the amplitudes of the road noise, river noise and the cricket songs using a GRAS microphone connected via a GRAS 12AX 4-channel power module and UltraSoundGate 816H data acquisition board to a laptop computer running Avisoft Recorder software, and then calibrated them using a ¼ inch free-field microphone [type 46BE, GRAS, Holt, Denmark; flat frequency response (±1 dB) from 100 Hz to 100 kHz] and calibrator (type 4231; Brüel and Kjær; 1 kHz tone, 94 dB SPL). Specifically, for this second calibration routine, we separately recorded each of (i) the same 3 min segment of road noise, (ii) the same 3 min segment of river noise and (iii) the same five repetitions of the full cricket song. As above and as in our previous experiments (Etzler et al., 2025a), the road noise, river noise and *T. commodus* song were played from the Bose speakers, while the *T. oceanicus* song was played from the Avisoft speakers. The ¼ inch microphone was placed at the same location as the starting position of the crickets and was pointed directly at the speakers (Avisoft Bioacoustics, Bose) which, as for behavioural and neural trials (see below), were 60 cm away.

Using the same equipment and settings, we also recorded a calibration tone (1 kHz, 94 dB SPL) using the Brüel and Kjær calibrator and used this as a reference in the Avisoft SASLab Pro software. Road noise and river noise measurements were taken from 36 sequential samples of 5 s duration. *Teleogryllus oceanicus* and *T. commodus* song measurements were taken from five repetitions of the song. For each sample, we measured the root mean square (rms) amplitude of the sound and the power spectrum as dB values per frequency relative to a maximum of zero. The rms amplitude of the calibration tone was used as a reference to determine the sound amplitude of each sample and the peak of each power spectrum was set to this amplitude with the amplitudes of the other frequencies being measured relative to peak [i.e. 94 dB SPL+20×log_10_(rms of sample/rms of calibration tone)+amplitude of frequency relative to peak]. We determined that road noise, river noise and cricket songs were on average 70 dB SPL rms at 60 cm from the speakers (see Fig. 1 for further details).

After the start of the acoustic playbacks, the focal cricket was given 60 s to acclimatise to its physical and acoustic environment before the plastic container covering the egg cup was removed, which marked the beginning of the trial. The observer (E.A.E.) was in an adjacent room separated by a door. We video recorded all trials at 15 frames per second using an HD 1080P CCTV dome camera (Avalonix, Buffalo, NY, USA) mounted 2.45 m overhead. Each trial lasted 5 min and was considered complete if the cricket had reached the edge of the circle or had failed to reach it by the 5 min mark. We scored crickets based on whether they successfully reached the focal speaker playing the male song (i.e. exiting from one of the two segments on either side of the focal speaker). For crickets that successfully exited the arena near the correct speaker, we determined how long they took to (a) emerge from the egg carton shelter (start latency). We then calculated (b) search time as the time from emergence from the shelter to the completion of the trial. To control for potential size-based variation in our statistical analyses, we weighed each cricket after the trial. We conducted all trials at ∼21°C.

Video footage of trials was further analysed using video tracking software (Ctrax v.0.5.18). We took a series of coordinates indicating the position of the cricket for each video frame from Ctrax and, using the R package ‘trajr’, used these coordinates to calculate the total distance travelled for each cricket, henceforth referred to as (c) path length. Following Schmitz et al. (1982), we also counted how often crickets paused during the search period, treating any period in which the cricket did not move for >7 video frames (i.e. over 0.53 s) as a single pause. The total of these pauses is henceforth referred to as the (d) pause number. Finally, we divided the path length (c) by the amount of time spent moving during search time (b) to get the (e) average speed of each cricket, where time spent moving was set as equal to (b) search time minus the time spent paused.

#### Auditory neurophysiology

To assess the possible impact of traffic noise on hearing, we recorded neural responses from 10 randomly selected crickets used in the behavioural phonotaxis trials. We pinned the crickets with their ventral side up to a block of modelling clay and removed the cuticle on the ventral surface between the head and thorax to expose cervical connectives containing axons of the AN1 and AN2 auditory interneurons. We used a hook-shaped tungsten recording electrode to hook the cervical connective while we placed a reference electrode in the abdomen. Electrodes were connected to a differential amplifier (DP-301, Warner Instruments, Hamden, CT, USA) and the output was connected to a data acquisition board (Avisoft Ultrasoundgate 816H, Avisoft Bioacoustics) for digital recording on a computer.

We used the same arena and speakers as described for the behavioural/phonotaxis trials, with minor modifications. There was no central shelter in the middle of the arena and we only used one pair of Bose speakers (which, as for the behavioural trials, were used only for noise treatments) that flanked a single Avisoft focal speaker (which was used only for cricket song in the behavioural trials but in this case played not only the song but also the 20 ms pure tones used to generate the audiograms in these neurophysiological trials). We placed the cricket neural preparation in the centre of this arena, 60 cm away from the focal speaker and with the ear ipsilateral to the recorded connective facing said speaker. We placed a microphone (CM16, Avisoft Bioacoustics) next to the neural preparation and facing the speakers, which recorded the playbacks on a separate channel of the same data acquisition board as the differential amplifier's neural trace output.

#### Audiograms

Only 9 of the 10 crickets performed the audiograms, with Q39 dying before the audiogram presentations. To make audiograms, we played increasingly loud pulses of sound at a range of frequencies (2–30 kHz in 2 kHz steps) from the focal Avisoft speaker. Frequencies were presented in random order. For each frequency, sound pulses increased in amplitude from 30 dB SPL to 80 dB SPL in 2 dB steps. Sound pulses were 20 ms in duration (including 1 ms rise/fall times). To avoid neural adaptation, we presented sound pulses at 500 ms intervals. In total, the time to complete each audiogram was 3 min and 8 s. The amplitudes of the audiogram sound pulses were calibrated using a ¼ inch free-field microphone (type 4939, Brüel and Kjær) and calibrator (type 4231; Brüel and Kjær). To do this, we played the sound pulses from the focal Avisoft speaker and recorded them using the Brüel and Kjær microphone, which was placed at the same location as the neural preparation and was pointed directly at the speaker with all other equipment in place. The microphone was connected via an Avisoft power module 40017 and an UltrasoundgateSG 116Hme data acquisition board to a laptop computer running Avisoft Recorder software. We also recorded a calibration tone (1 kHz, 94 dB SPL) using the same equipment and settings as a reference to adjust the sound pulses to the correct amplitudes using Avisoft SASLab Pro software. Sound pulses were recorded again after calibration to ensure they were the correct amplitude in the setup. Audiograms were run three times, once each with silence, road noise and river noise as background acoustic treatments. Road and river noise both peaked at ∼1 kHz and were calibrated to be the same amplitude at the cricket as used for the centre of the arena in behavioural experiments (∼70 dBA). We determined that road noise, river noise and cricket songs were all on average 70 dB SPL rms at 60 cm from the speakers (see Fig. 1 for further details).

We did not use *T. commodus* song as a background noise treatment when making our audiograms because we suspected that interneuron activity would be high in response to this background noise treatment, making it too difficult to determine the threshold response to the sound pulses that comprise the audiogram test. *Teleogryllus commodus* song has a carrier frequency of 4 kHz, which is higher than the dominant frequencies in river and traffic noise (<1 kHz in our samples; Fig. 1), and within the range of frequencies to which *T. oceanicus* performs positive phonotaxis (Moiseff et al., 1978). Thus, testing the threshold of response at 4 kHz and 6 kHz would thus have been near impossible given that the stimulus and the background treatment would have been very similar.

#### Neural responses to *T. oceanicus* song

To measure auditory interneuron activity in response to conspecific male song under all four background acoustic treatments, we played *T. oceanicus* song from the focal speaker and background noise treatments (silence, traffic noise, river noise or *T. commodus* song) from the flanking speakers to the 10 cricket neural preparations. Background acoustic treatments were calibrated to be the same amplitude as the cricket experienced in the centre of the arena for the behavioural phonotaxis trials (∼70 dB SPL). We first recorded neural activity in the background noise treatment for 1 min to establish a baseline response to said background. After 1 min, we began playback of the optimized *T. oceanicus* song from the focal speaker alongside the background noise treatment for an additional minute. Crickets were exposed to the four treatments in random order.

As for the road noise, river noise and cricket song playbacks used in our phonotaxis/behavioural experiments, we also calculated the average dB SPL rms amplitude of the 3 min segment of road noise, 3 min segment of river noise and five cricket song sequences for both *T. commodus* and *T. oceanicus* at the neural preparation and, as for the behavioural trials, determined them to be ∼70 dB SPL rms. That is, we did so as we have described for the behavioural trials, except we used a Brüel and Kjær ¼ inch microphone rather than a GRAS ¼ inch microphone. Both microphones have a flat frequency response (±1 dB) between 100 Hz and 100 kHz.

### Data analysis

#### Behaviour

We used a Chi-squared test to determine whether the movement of crickets leaving the arena was likely to be due to random chance, and whether the number of crickets successfully reaching the focal speaker differed based on the noise playback treatment. Then, using just the data for the crickets that successfully reached the correct speaker, we used linear models to examine the effect of noise playback treatment on the measured behavioural variables, using traffic noise as the reference level for *post hoc* comparisons. A single cricket was excluded from this analysis because it took an order of magnitude longer to search the arena than the others. Models included noise playback treatment as a predictor, and insect mass and room temperature as control variables. Models had one of the following as the response variable: (a) start latency, (b) search time, (c) path length, (d) number of pauses or (e) average speed. The residuals for search time and start latency were found to be non-normal using a Shapiro–Wilk test and so were log transformed to correct for skew in the data. The model for number of pauses used a Poisson family link function. Pairwise comparisons for the number of pauses model were done using Tukey HSD test on the effective marginal means.

#### Audiograms

We analysed the recordings visually using sound analysis software (SASLab Pro, Avisoft Bioacoustics) but were unable to reliably identify AN1 spikes well enough to count them. Instead, we looked at the relative AN1 activity before and after a sound pulse. For each audiogram, we recorded the start time of each pulse in the 4, 6 and 30 kHz sequences. We chose these frequencies based on 4 and 6 kHz being close to the frequency of *T. oceanicus* song (∼5 kHz) and 30 kHz being a frequency that would elicit AN2 spikes but not AN1.

In Avisoft SASLabPro, we band-pass filtered the neural recordings from 300 Hz to 10 kHz (zero phase, Hamming Window, 1024 taps). AN2 spikes were then identified using the Pulse Train Analysis feature and labelled. We then used the Change Volume feature to reduce the amplitude of these spikes to zero. We used a custom R script to measure the rms amplitude of the neural recordings from 40 ms before to 10 ms after the start of each sound pulse (50 ms pre-latency) and from 10 ms to 60 ms after the start of each sound pulse (50 ms post-latency). Sections that contained significant neural noise that prevented the identification of spikes were excluded from further analysis. We then calculated the amplitude difference in dB pre- and post-latency [20×log(post-latency RMS/pre-latency rms)] and then ran a repeated measures ANOVA to compare the effect of background noise treatment on the delta rms amplitude, for each amplitude level in the audiogram at each frequency. Bonferroni corrections were then applied to correct for the number of tests.

We identified AN2 spikes using their high-frequency sensitivity and large spike amplitude relative to other neural activity in the recordings. Using the unmodified recordings, we measured AN2 threshold (dB SPL) for each frequency as the lowest amplitude sound pulse to elicit a burst of AN2 spikes. We classified it as a burst/response to the sound pulse if (i) the latency of the first spike was less than 50 ms after the start of the sound pulse, and (ii) more than one spike was present with intervals <20 ms between them. These values were selected because they represent maximum values for published data of AN2 cell recordings in *T. oceanicus* (Faulkes and Pollack, 2000; Samson and Pollack, 2002). We did this for recordings made in silence or with background noise (traffic or river). We first ran a mixed effect linear model with amplitude at threshold as the response variable, noise playback treatment and frequency of the tone as fixed effects and cricket ID as a random effect. Based on the results of the linear model, we then ran within-subject mixed ANOVA with the amplitude at threshold as the response variable for each frequency in the audiogram and noise playback treatment as a factor in the model. Because of the number of tests (14), we applied Bonferroni correction (Rice, 1989).

#### AN1 responses to *T. oceanicus song*

Individual AN1 action potentials could not be identified reliably in the recordings to count spikes. However, for each treatment, we had many recordings of responses to male song sound pulses. Therefore, we took the rectified average of many repetitions to visualize the AN1 response to male song pulses.

We analysed recordings of neural activity in response to the *T. oceanicus* song playback for three of the acoustic background treatments: silence, river noise and road noise. One minute of song was played for each treatment. *Teleogryllus oceanicus* females show a strong preference for chirp pulse repetition rate, but no strong preference for trill rate. Likewise, females will perform phonotaxis to songs consisting only of chirps, but not to song of only trills (see references in Gerhardt and Huber, 2002). Therefore, we measured the neural response to individual pulses of the chirps.

Noise playbacks consisted of 1 min of background noise before 1 min of *T. oceanicus* song was added to the background sound. The song was a repeating 1.3 s sound pulse sequence consisting of 12 chirp pulses (30 ms duration, 60 ms period) and four trills (doublets of pulses). We trimmed each recording in SASLab Pro to include chirps 11–40. We excluded the first 10 chirps of song playback to restrict measurements to those after the neural activity had adapted to the stimulus.

Recordings were down sampled from 300 kHz to 48 kHz sampling rate to reduce processing time. Files were high-pass filtered at 300 Hz to remove electrical noise. The start time of the first pulse of the first chirp was measured for calculating the times of the rest of the chirp pulses. Because the AN2 neuron also responds to song pulses, we used the Pulse Train Analysis feature to label the AN2 spikes for each recording. The section labels mark the start and end of the AN2 spikes. We then used the Change Volume feature to make the amplitude of the AN2 spikes zero.

We used a custom R script to extract the sample values for 60 ms from the start of each chirp for both the neural activity (channel 1) and the sound (channel 2) during this time. The data were rectified (absolute values calculated) and the average value for each sample in the 360 repetitions was calculated. The start time was standardized by finding the time 20 ms before the peak of the first chip pulse in the 11th chirp.

Three crickets (Q36, Q39 and Q42) were excluded because AN2 was not reliably greater in amplitude than the other neural activity and therefore could not be excluded by a threshold in the Pulse Train Analysis feature. Therefore, measurements were taken from 7 crickets.

#### AN2 responses to *T. oceanicus* song

To assess neural responses to *T. oceanicus* song, we counted the number of AN2 spikes in a 1.3 s period at three time points during the song recordings: (i) 30 s after the background noise treatment had begun (silence, traffic noise, river noise or *T. commodus* song), (ii) at the start time of the *T. oceanicus* song, and (iii) 30 s after the *T. oceanicus* song had started. We chose 1.3 s as it is the duration of the repeated pulse sequence of the *T. oceanicus* song. For each time point, we ran linear mixed models with the number of AN2 spikes as the response variable, noise playback treatment as the main effect, and individual crickets as random effects. Type III Wald Chi-square tests were used to estimate *P*-values from the linear models. At each time point, we also measured the minimum instantaneous spike rate for AN2 (inverse of time between two spikes). We then ran linear mixed models with minimum instantaneous spike rate as the response variable, noise playback treatment as the main effect, with individual cricket as a random effect. Type III Wald Chi-square tests were used to estimate *P*-values from the linear models.

## RESULTS

### Behaviour

Sixty-four of the 96 crickets tested reached the correct (i.e. song-broadcasting) speaker. Thirty of the 32 crickets that failed to reach the correct speaker did not even leave the central shelter. The remaining two left the arena from a segment not abutting the focal speaker. These observations differ significantly from the movement distributions expected by random chance (χ^2^=176.33, *P*<0.0001). Thus, we are confident that the movement we observed was indeed positive phonotaxis. We also found that background noise treatment did not significantly impact whether the crickets successfully reached the song-broadcasting speaker or not (χ^2^=1.39, *P*=0.239). Only the 64 crickets that reached the correct speaker were included in the analysis. One individual was then excluded as an extreme temporal outlier, leaving a final total of 63 crickets.

**Fig. 3.**
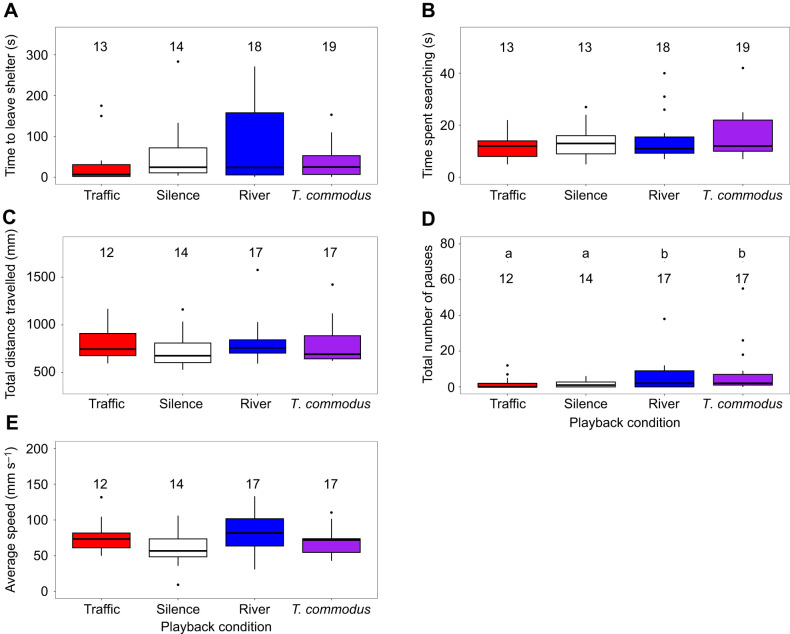
**Results of phonotaxis experiments for female crickets listening to male song in a background of silence, traffic noise, river noise or male song from *T. commodus*.** (A) Time from the start of the experiment to when the female left the central shelter, (B) time from when the female left the shelter until she reached the speaker, (C) total distance travelled by the female, as measured by the path length, (D) total number of times that the female paused walking for more than 0.5 s and (E) average speed of walking, measured as the total path length divided by the time spent walking. Data are shown as box plots, where the box represents the interquartile range, the line in the box represents the median and whiskers are Q1/Q3+1.5×interquartile range. Outliers are shown as points. Letters above bars indicate significant differences (D: linear model, *P*<0.05); no letters indicate no significant differences (A–C,E). Sample size for each condition is listed above each bar.

When compared with traffic noise, the other three acoustic treatments did not significantly affect the crickets' (a) time to leave the shelter (Table 1, Fig. 3A), (b) search time (Table 1, Fig. 3B), (c) total distance travelled (Table 1, Fig. 3C) or (e) average speed (Table 1, Fig. 3E). However, the number of pauses did differ (Table 1, Fig. 3D). Crickets paused significantly more often in the river noise and *T. commodus* treatments than in the traffic noise treatment, although this had little impact on search time. This is likely due to individual pauses being fairly short in length (time spent paused: mean=1.226 s, median=0 s, range=0–21 s), compared with the time spent searching (time spent searching: mean=14.254 s, median=11 s, range=5–42 s).

**Table 1.** Linear model testing the effect of background noise playback treatment on time to leave the shelter, time spent searching, total path length, number of pauses and average speed

Characteristic	Time to leave shelter			Time spent searching			Total path length			Number of pauses			Average speed		
	β	95% CI	*P*	β	95% CI	*P*	β	95% CI	*P*	log(IRR)	95% CI	*P*	β	95% CI	*P*
Treatment			0.6			0.8			0.8			<0.001			0.4
Traffic	–	–		–	–		–	–		–	–		–	–	
River	0.22	−0.28, 0.72	0.4	0.04	−0.12, 0.19	0.6	31	−127, 188	0.7	0.83	0.41, 1.3	<0.001	5.0	−13, 23	0.6
Silence	0.21	−0.33, 0.75	0.4	0.02	−0.15, 0.19	0.8	−48	−216, 121	0.6	−0.35	−0.94, 0.23	0.2	−10	−30, 9.3	0.3
*T. commodus*	−0.03	−0.55, 0.48	0.9	0.08	−0.08, 0.24	0.3	15	−149, 179	0.9	1.1	0.68, 1.6	<0.001	−4.4	−23, 15	0.6
Mass (g)	2.5	0.34, 4.6	0.024	0.23	−0.42, 0.88	0.5	43	−615, 700	0.9	6.9	5.5, 8.3	<0.001	11	−65, 87	0.8
Temperature (°C)	−0.17	−0.29, −0.06	0.003	−0.01	−0.04, 0.03	0.7	25	−9.2, 60	0.15	−0.04	−0.14, 0.06	0.4	2.1	−1.9, 6.1	0.3

CI, confidence interval; IRR, incidence rate ratio. Treatment, mass and temperature were all modelled as fixed effects. Overall treatment *P*-value is from a type II ANOVA, and traffic noise was used as the reference value in the linear model.

### Neurophysiology

#### Audiograms

**Fig. 4.**
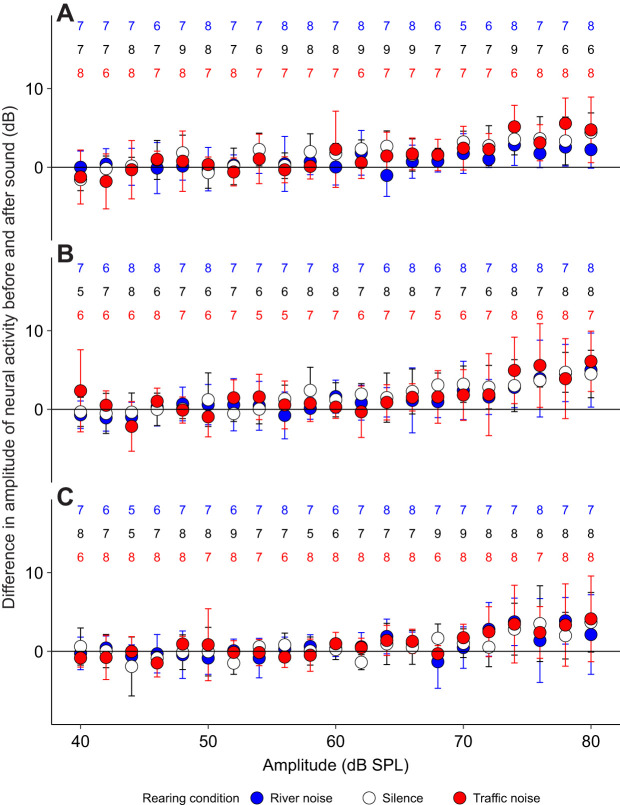
**Difference in root mean square (rms) amplitude of neural activity before and after sound pulses of different frequencies.** (A) 4 kHz; (B) 6 kHz; (C) 30 kHz. AN2 action potentials were removed prior to rms measurements. Values are means, error bars are s.d. Sample size for each condition is shown above in the associated colour.

For AN1 rms values, we found no significant differences between treatments after Bonferroni correction, indicating that background noise treatment had no effect on AN1's ability to respond to signals in the environment (Fig. 4) (Table 2).

**Table 2.** Summary table of repeated measure ANOVA for AN1 root mean square (rms) values for the audiograms

Frequency (kHz)	Amplitude (dB SPL)	*F*-statistic	*P*	*P*_adj_	d.f.
4	40	0.874	0.442	1	12
4	42	2.085	0.187	1	8
4	44	0.227	0.801	1	10
4	46	1.342	0.358	1	4
4	48	0.65	0.540	1	12
4	50	0.45	0.65	1	10
4	52	1.026	0.401	1	8
4	54	0.111	0.897	1	6
4	56	0.341	0.718	1	12
4	58	2.647	0.12	1	10
4	60	2.025	0.194	1	8
4	62	0.679	0.529	1	10
4	64	3.786	0.06	1	10
4	66	0.24	0.790	1	12
4	68	1.552	0.317	1	4
4	70	0.367	0.708	1	6
4	72	0.246	0.793	1	4
4	74	1.725	0.227	1	10
4	76	2.120	0.171	1	10
4	78	1.956	0.203	1	8
4	80	4.078	0.06	1	8
6	40	1.079	0.422	1	4
6	42	1.979	0.219	1	6
6	44	0.740	0.507	1	8
6	46	0.483	0.634	1	8
6	48	0.317	0.737	1	8
6	50	0.407	0.683	1	6
6	52	1.137	0.359	1	10
6	54	0.423	0.673	1	6
6	56	0.259	0.784	1	4
6	58	3.307	0.09	1	8
6	60	0.409	0.675	1	10
6	62	3.234	0.111	1	6
6	64	4.241	0.055	1	8
6	66	0.657	0.54	1	10
6	68	1.41	0.315	1	6
6	70	2.309	0.180	1	6
6	72	0.585	0.580	1	8
6	74	0.981	0.403	1	12
6	76	0.333	0.726	1	8
6	78	0.024	0.976	1	12
6	80	0.118	0.89	1	10
30	40	0.114	0.894	1	8
30	42	1.703	0.242	1	8
30	44	0.803	0.509	1	4
30	46	0.404	0.685	1	6
30	48	1.381	0.295	1	10
30	50	0.421	0.668	1	10
30	52	2.22	0.159	1	10
30	54	3.375	0.104	1	6
30	56	0.330	0.731	1	6
30	58	0.803	0.491	1	6
30	60	0.097	0.90	1	6
30	62	3.502	0.081	1	8
30	64	0.155	0.859	1	10
30	66	0.105	0.902	1	10
30	68	2.013	0.196	1	8
30	70	0.414	0.670	1	12
30	72	0.280	0.762	1	10
30	74	0.719	0.511	1	10
30	76	1.641	0.242	1	10
30	78	0.869	0.449	1	10
30	80	4.715	0.036	1	10

Repeated measure ANOVA had treatment as the explanatory variable and pre- and post-latency rms difference (measured in dB), for each level of frequency and amplitude. Adjusted *P*-values (*P*_adj_) were corrected using Bonferroni corrections.

**Fig. 5.**
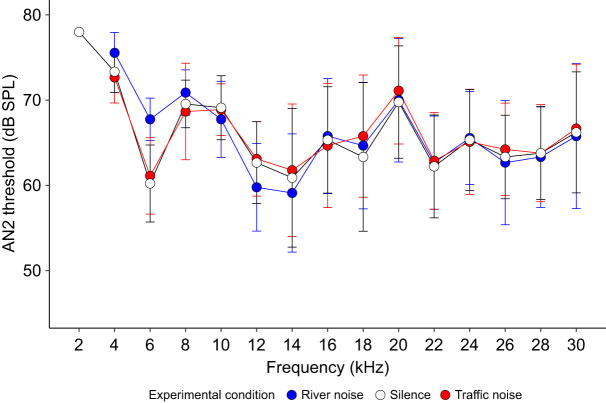
**AN2 response thresholds of female crickets at sound frequencies between 2 and 30 kHz.** Sound pulses at different frequencies were played back in silence (white circles), traffic noise (red circles) and river noise (blue circles). When tested in river and traffic noise, crickets did not show any response at 2 kHz. The silent and traffic noise audiograms were previously published in Etzler et al. (2025a). Values are means, error bars are s.d. Sample size was 9 for each mean.

For AN2 thresholds under background treatments of silence, traffic noise or river noise, we found a significant interaction effect between background noise treatment and the frequency (kHz) of the sound pulse (*F*=2.07, *P*<0.01, d.f.=156). We then performed an ANOVA for each presented frequency from 4 to 30 kHz and found no significant difference between these treatments after Bonferroni correction except at 6 kHz (Fig. 5) (Table 3). At 6 kHz, the AN2 threshold was significantly higher in the river noise treatment compared with the traffic noise treatment, though traffic noise and silence did not significantly differ from each other. The 6 kHz frequency is of particular interest as the carrier frequency of the calling song of *T. oceanicus* is ∼5 kHz, which indicates that river noise would be most likely to interfere with phonotaxis. While we did not note any difference in the crickets' behaviour, the 6 kHz tone threshold was at ∼68 dB SPL, slightly below the amplitude of the calling song played during the behavioural trials. We therefore acknowledge that there might have been an observed negative effect of river noise on phonotaxis had there been a greater difference in amplitude between the noise and signal at this and other overlapping frequencies.

**Table 3.** ANOVA results for the effect of background noise treatment on AN2 audiograms of female crickets for sound frequencies between 4 and 30 kHz

Frequency (kHz)	*F*-statistic	*P*	d.f.
4	5.478	**0.015**	16
6	16.011	**<0.001**	14
8	1.848	0.190	16
10	0.655	0.535	14
12	5.727	**0.013**	16
14	5.671	**0.014**	16
16	0.319	0.731	16
18	0.692	0.515	16
20	0.597	0.564	14
22	0.206	0.816	16
24	0.045	0.956	16
26	0.320	0.731	16
28	0.125	0.884	16
30	0.283	0.757	16

Significant results pre-Bonferroni correction are marked in bold; see Results for details.

#### Responses to *T. oceanicus* song

**Fig. 6.**
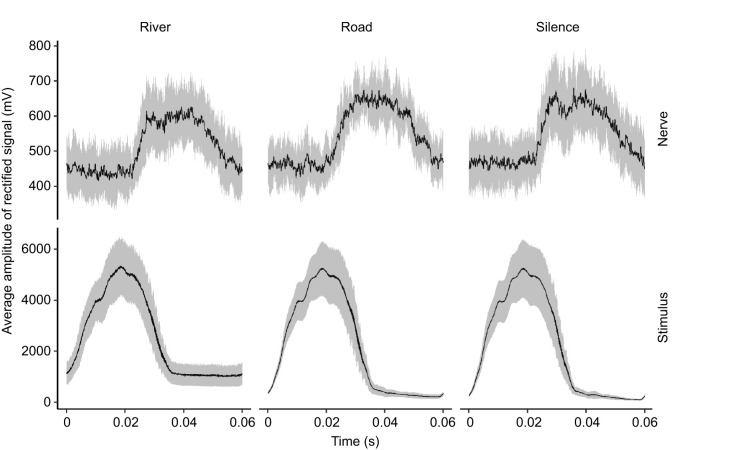
**Rectified average of neural and stimulus recordings for 7 crickets presented with *T. oceanicus* song alongside a background noise treatment.** An average was taken over 360 chirps. AN2 action potentials were removed prior to averaging.

The AN1 response to chirps is apparent when the average data for all crickets is plotted (Fig. 6). Two variables were measured from the averages: area under the curve for the AN1 response (0.02–0.06 s) and the latency, estimated by finding the breakpoint for data in the range 10–30 ms. Linear mixed models with cricket ID as a random effect did not show significant differences across treatments for these two variables (area: *F*=1.1, *P*=0.372; breakpoints/latency: *F*=0.9, *P*=0.419).

**Fig. 7.**
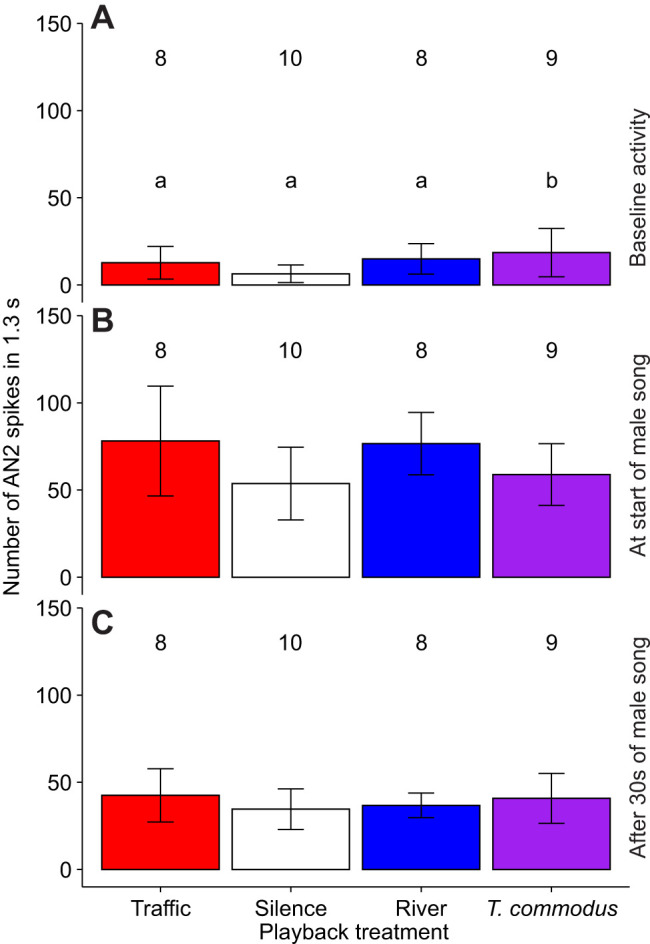
**Number of AN2 spikes in 1.3 s under three treatments.** (A) Baseline activity in silence or traffic noise. (B) Activity during the first 1.3 s of male song. (C) Activity after continuing to hear male song for 30 s. Values are means, error bars are s.d. Different letters above bars indicate significant differences (linear model, A: *P*<0.05); no letters indicate no significant differences (B,C). Sample sizes are indicated by the numbers above the bars.

For AN2 spike counts, we found a significant effect of background acoustic treatment before the playback of *T. oceanicus* song (*F*=3.5, *P*=0.039, d.f.=18), where the AN2 activity was significantly higher in the *T. commodus* song treatment (*P*=0.010; Fig. 7A). We found no significant difference between all four background acoustic treatments immediately after the onset of male song (*F*=2.0, *P*=0.149, d.f.=18) (Fig. 7B), or after 30 s of continuous male song (*F*=1.9, *P*=0.16, d.f.=18) (Fig. 7C). We did, however, find a significant effect of background acoustic treatment on instantaneous spike rates immediately after the onset of male song (*F*=3.2, *P*=0.041, d.f.=24). Pairwise *t*-tests showed that the instantaneous spike rates immediately after the onset of male song in the *T. commodus* treatment were significantly lower than those in the river treatment (*P*=0.011).

## DISCUSSION

We found no support for the hypothesis that traffic noise inhibits cricket acoustic communication, either through inhibiting detection or localization, compared with naturally occurring noises. At the level of behaviour, we found instead a significant difference in the number of times female crickets paused during phonotaxis to conspecific male song, but in the opposite direction to that which would be predicted if traffic noise impeded phonotaxis and related behaviours. Specifically, under our experimental treatments, we found crickets paused more often in the presence of natural background sounds (river noise and *T. commodus* song) than they did in traffic noise, and that pause number did not differ between silence and traffic noise (Fig. 3D). The crickets in our experiment experienced song amplitudes ranging from 70 to 92 dB SPL as they approached the speaker, which are high as crickets typically produce song with source levels of 90 dB SPL at 10 cm (Balakrishnan and Pollack, 1996). With respect to AN1, we found no effect of background noise treatment on either the audiograms or the response to song. We found that significantly higher amplitudes were required for AN2 to respond to sound pules of 6 kHz during river noise compared with both silence and road noise (Fig. 5). AN2 spike rates were, as predicted, highest in the *T. commodus* song background noise treatment before the *T. oceanicus* song stimulus was presented (Fig. 7A). After the *T. oceanicus* song presentation, instantaneous spike rates were lowest in the *T. commodus* song background noise treatment, but this result was only significantly different from the river noise background treatment.

As crickets pause during sound localization to re-orient themselves (Murphey and Zaretsky, 1972), one plausible interpretation of our results is that our adult female crickets in the river and *T. commodus* background acoustic treatments needed to check their bearings more often to localize the conspecific male song source than they did in silence or traffic noise. This could be due to masking effects as both river noise and *T. commodus* song have more energy in frequencies close to or overlapping with *T. oceanicus* song than do either road noise or silence (Fig. 1). Specifically, all else being equal, river noise is >20 dB louder than road noise at frequencies above 3 kHz (Fig. 1). The difference in the number of pauses, however, did not reduce the likelihood of crickets reaching the correct speaker or the time spent searching between the four background acoustic treatments. This is likely due to the small length of time spent paused relative to total search time (search time: 15.7±14.5 s; time spent paused: 2.4±4.2 s; means±s.d. with all treatments and crickets pooled). Likewise, for those crickets that successfully reached the correct speaker, the background acoustic treatment did not significantly affect latency to leave the shelter, distance travelled or walking speed (Fig. 3A,C,E).

Our neural results also support the idea that masking by river noise could be slightly negatively influencing the crickets' searching behaviour. The AN2 audiograms made in background acoustic treatments of silence, traffic noise or river noise revealed only a difference in hearing thresholds between treatments at 6 kHz, one of the two frequencies we tested that is closest to the 5 kHz carrier frequency of *T. oceanicus* song (Fig. 5). Specifically, at 6 kHz, we found that a significantly louder tone was required to elicit AN2 activity during the river noise treatment compared with silence or road noise, indicating that river noise made 6 kHz tone detection more difficult. We also found that the number of AN2 spikes per 1.3 s significantly differed between noise treatments before the start of *T. oceanicus* song, with more AN2 spikes during the *T. commodus* song treatment (Fig. 7). At the onset of *T. oceanicus* song however, the *T. commodus* treatment elicited a significantly lower instantaneous spike rate than did the river noise treatment, possibly due to neural adaptation. However, none of this applies to those AN1 responses we were able to detect. Given that AN1 is the primary interneuron responsible for the detection of conspecific song, it is unclear whether the weak effects we found for AN2 would have any fitness consequences at all.

Much has been made of how anthropogenic noise can interfere with animal acoustic communication, yet little attention has been paid to whether those dangers are in fact a novel source of selection or merely a variation on pre-existing natural sources of noise (Gomes et al., 2021). Our results indicate that, compared with silence, river noise has a greater effect on cricket phonotaxis than traffic noise, despite having been present throughout the evolutionary and ecological history of *T. oceanicus*. In fact, many abiotic ambient noises, such as wind and rain, are like traffic noise in having most energy at frequencies lower than that of the *T. oceancius* song (Sánchez-Giraldo et al., 2020). This would suggest that the relative novelty of traffic noise does not necessarily mean that crickets would be poorly adapted to compensate for it. Indeed, crickets have been shown to be excellent at dealing with auditory distractions. Crickets that sing in acoustic environments with many heterospecific songs of similar frequency, including *T. oceanicus*, often have narrowly tuned auditory interneurons that allow for reproductive isolation (Bailey et al., 2017; Morley and Mason, 2015; Römer and Bailey, 1998; Schmidt et al., 2011; Schmidt and Balakrishnan, 2015; Schmidt and Römer, 2011). The mechanisms used to distinguish between different cricket species' songs may well act in distinguishing song from background noise (Raboin and Elias, 2019).

Many orthopteran species, including our study species, are found near roads (Welsh et al., 2023). Assemblage surveys have found that while roads are aversive to some species, they attract others (Bhardwaj et al., 2019; Rebrina et al., 2022a,b; Sergeev, 1998; Torma et al., 2017). This may explain why some roadside habitats are seemingly better at preserving biodiversity and species richness than neighbouring agricultural land (Jakab and Nagy, 2022). Species differences may explain some of the variation in response to traffic noise. For example, *Oecanthus pellucens* is found more frequently along roadside ditches than further away (Rebrina et al., 2022a,b; Torma et al., 2017) and their acoustic communication seems to be unaffected by traffic noise (Costello and Symes, 2014; but see Orci et al., 2016), despite the relatively low fundamental frequency of their song (2.0–3.7 kHz; Orci et al., 2016), which is closer to the predominant frequencies in traffic noise than that of *T. oceanicus* (5 kHz; Bailey and Macleod, 2014). However, for these species and others, it will likely often prove difficult to disentangle the effects of traffic noise from those of vegetation differences between road edges and field on habitat preference.

*Teleogryllus oceanicus* almost exclusively lives in disturbed grassland near coasts (Zuk et al., 2001) and has likely evolved in the presence of repetitive low-frequency ocean waves. Many other cricket species (e.g. *Gryllus pennsylvanicus*) produce songs at 5 kHz and live near rivers (Harrison et al., 2013; Münsch et al., 2013; Rand and Harrison, 1989), which suggests they might be less affected by this noise during phonotaxis. It would be interesting to compare the songs, phonotactic behaviour and interneuron responses of cricket species that live near rivers with those of *T. oceanicus* to examine whether there are biologically meaningful differences in song frequencies or auditory filtering processes. Given the difficulty in finding even moderate deleterious effects of anthropogenic noise in *T. oceanicus*, a well-studied species in this context (Etzler et al., 2025a; Gurule-small and Tinghitella, 2018, 2019; Welsh et al., 2023), it will be important to examine whether *T. oceanicus* is uniquely resilient to anthropogenic noise compared with other orthopterans.

In conclusion, we found that under our experimental treatments, anthropogenic and natural noises did not have a deleterious effect on phonotaxis in *T. oceanicus*. Although natural noises might require crickets to pause more to gain directional information, this did not affect the total time needed by the cricket to locate the source of male song. The effects of river noise and heterospecific song highlight the need to compare traffic noise against appropriate controls. For example, to demonstrate fitness consequences of anthropogenic noise, it would need to be demonstrated that crickets are performing worse in the presence of anthropogenic noise relative to natural background noises, such as rain, wind, rivers or acoustic signals from other species, not just quiet laboratory treatments. What is considered a natural relevant background noise will vary with the wide variety of habitats that orthopterans use, within and across species. We expect a correlation between a given species and its natural environment's soundscape, with species that are more likely to encounter noisy environments better able to filter noise (e.g. Schmidt et al., 2011). In sum, the extent to which anthropogenic noise interferes with communication is likely to be species and habitat specific, and is an important consideration for future conservation efforts.
